# Screening mammography with special reference to guidelines in South Africa

**DOI:** 10.4102/sajr.v22i2.1370

**Published:** 2018-10-31

**Authors:** Shirley Lipschitz

**Affiliations:** 1 Dr Shirley Lipschitz and Associates, Sunninghill, Sandton, South Africa

## Abstract

Screening mammography is known to reduce mortality from breast cancer. Controversy regarding screening has led to much confusion in the medical fraternity. The purpose of this review is to point out the ‘pros and cons’ of screening. The benefits and perceived harms of screening will be discussed using evidence-based literature from the past 30 years. The literature was obtained from various journals sourced from the Internet. General findings are that screening mammography from the age of 40 saves lives, but that the problem of overdiagnosis and overtreatment of certain breast cancers overrides the benefit of screening. The article also covers the debate on what age to begin screening. Screening in the South African context is discussed. Screening in the future will need to be more selective of patients and of which cancers to treat less aggressively, if at all.

## Introduction

Screening mammography by means of X-ray imaging studies is the universal standard used to locate preclinical breast cancers. There is strong evidence that mammography screening results in a reduction of breast cancer deaths, principally through the effect of reducing the incidence of advanced and inoperable breast cancer with metastases.^1^ Screening leads to less morbidity and more effective treatment. It has been shown that a lower histological grade, smaller tumour size and absence of lymph node disease are factors associated with a better prognosis.^1^ Localised disease is associated with a 98.6% five-year relative survival rate, as compared with an 84.9% rate for cancers with lymph node positivity and a 25.9% rate for those with metastases.^2,3^

Proponents and detractors of screening have different interpretations and extrapolations from evidence-based statistics and this has caused confusion with regards to screening recommendations in particular. Different countries, medical societies, referring physicians and funders have varying guidelines for screening mammography.

The following review of some of the literature includes those studies that concern the benefits and disadvantages of screening, the controversies of screening in the 40–50 age group and the issues around screening intervals. Reference will also be made to screening in the South African context.

## The benefits of screening

Evidence-based studies have shown that screening mammography saves lives.^1,2,3^ This has been demonstrated through randomised control trials (RCTs)^4,5^ and numerous observational studies.^6,7,8,9,10^

A meta-analysis of RCTs has shown a 15% – 30% reduction in breast cancer mortality in the 40–74-year-old age group who were invited to screening. The RCT with the longest follow-up (29 years) is the Swedish Two-County Trial, which was the first trial to demonstrate a breast cancer mortality reduction associated with an invitation to mammographic screening without clinical breast examination.^1^ However, these historical RCTs may not be relevant today because of the new screening technologies and reported bias in the trials (as certain women invited to screening were not compliant, while other women in the control group had opportunistic screening).

Case-control studies, cohort studies and trend analyses have been used to evaluate the effects of screening. Gabe and Duffy^6^ summarised ways of evaluating screening in a non-experimental setting, as well as the findings from 38 non-randomised studies on breast cancer screening. Their study results showed breast cancer mortality reductions in the order of 30% – 40% associated with screening.

Broeders et al.^7^ summarised the findings from the evaluations of modern, organised mammography programmes in Europe. They showed a 25% decrease in mortality with those invited to screening and a 38% reduction in those actually exposed to screening in a meta-analyses of the incidence-based mortality studies.

The Norwegian breast cancer screening programme began in 1996 with biennial screening of the 50–69-year-old group. Breast cancer mortality was assessed among women who were exposed and not exposed to screening between 1996 and 2009, and followed for breast cancer death through to 2010 (average follow-up was 5.7 years). A 43% mortality reduction was shown after 15 years of follow-up. The results in this follow-up were adjusted for age, years in the programme and selection bias.^8^

Coldman et al.^9^ showed, in the Pan-Canadian Study of Mammography Screening and Mortality from breast cancer, lower mortality rates in participants of the trial, compared with expected rates among non-participants. Those who participated in the trial at 40–49 showed a 44% decrease in mortality, those in the 50–59 group showed a 40% decrease, those in the 60–69 age group showed a 42% decrease and those in the 70–79 age group showed a 35% decrease. The average reduction in mortality in all age groups and provinces was 40% in the exposed group.

The Cancer Intervention and Surveillance Modelling Network (CISNET)^10^ assessed that screening and treatment contributed to a 46% reduction in breast cancer deaths over the 1975–2000 period in the United States, and later estimated that if there was full compliance with screening and treatment, there would have been a 68% – 74% reduction in mortality.

In summary, the RCT and observational trials have shown a significant decrease in mortality rates in those screened. Possible reasons why greater reductions have been reported in observational studies, compared with RCTs, include bias, improved treatment of early-stage breast cancer, improved screening technology and a difference between actual participation versus invitation. Those opposed to screening believe RCTs were biased and observation trials do not allow for the positive impact of new treatments.

## The harms of screening

Those against screening believe that it has led to overdiagnosis and overtreatment, and that there has not been a sufficient reduction in late-stage disease to justify screening.^11^ The cost and benefit of lives saved versus the harms of screening and potential overdiagnosis are the core issues underpinning the controversies raging for the past 10–15 years. Funding of screening is significant and economic considerations may have an impact on decision-making.

Screening leads to an average recall rate of around 10%.^12^ These women are usually recalled for extra views and sonography. Most of these women are subjected to minor, mostly psychological stresses. Some of these women (1% – 2%) need a biopsy, which can lead to the concept of false positivity before the results reveal otherwise. The extra stress and funding for these tests are perceived as harms of screening. Furthermore, cumulative radiation exposure is thought by many to be harmful. However, radiation doses for each mammogram are well below acceptable limits, while the real impact of years of mammography on an individual cannot be readily quantified and is only estimated.^11^

Overdiagnosis of a cancer is a perceived harm of screening. The commonly accepted definition is that of a diagnosed preclinical cancer, which would not have led to a life-threatening situation, had it not been detected on mammography. Screened women are diagnosed with breast cancer before unscreened women because of lead time, which is defined as the time between the screening-detected cancer and the time it would present clinically. Only a long-term follow-up between screened and unscreened women would correct the issue of perceived overdiagnosis. Older women, already screened, would presumably have fewer cancers based on their long-term history of screening. It is generally thought that lead time results in an overestimation of overdiagnosis.^12^ The counterargument to the overdiagnosis theory^13^ is that screening picks up more cancers because of lead time and this should not be termed overdiagnosis.

Estimates of an overdiagnosis rate of 1% – 10% with screening mammography are generally accepted as the benchmark.^12^ The EUROSCEEN (European breast cancer service screening) group has calculated that with proper adjustment for known confounders, the most accurate estimate of overdiagnosis ranged from 1% to 10%. Long-term follow-up of RCT data suggests that overdiagnosis is mostly because of ductal carcinoma *in situ* (DCIS).^14^

Most of the papers on the topic have been performed by epidemiologists and showed a wide variation of reports of overdiagnosis, ranging from 0% to 30%.^15^ Bleyer and Welch^16^ collected data from the US National Cancer Institute’s Surveillance Epidemiology, and End Results (SEER) Program. They said that the imbalance between early-stage and late-stage breast cancers between the pre-screening era (1976–1978) and the current era (2006–2008) shows that screening led to overdiagnosis. The accuracy was questioned as they also did not account for lead time and underestimated the background cancer incidence rate in the 30-year period they were observing. Marmot et al.^17^ used three RCT late follow-up studies of mainly older women, with both *in situ* and invasive disease, to estimate the approximate rate of overdiagnosis as 10.7% and also postulated a 1% lifetime risk of overdiagnosis in women screened between the ages of 50 and 70. In an age trial in the United Kingdom (UK), Gunsoy et al.^14^ showed a rate of overdiagnosis of 0.7%. Helquist et al.^18^ in Sweden reviewed 3.8 million people aged 40–49 and found an overdiagnosis rate of 1%.

In a currently published trial by Hendrick,^13^ SEER data were used to calculate overdiagnosis rates based on lead time estimations of 24 months for pre-menopausal women and 40 months for post-menopausal women. This showed the general overdiagnosis for DCIS to be 9%, and for invasive and other tumours to be 7%. However, in the 40–49 age group, overdiagnosis rates were the lowest, being 0.15% for DCIS and below 0.1% for invasive and other breast cancers. Overdiagnosis rates increased progressively with age. The Cancer Intervention and Surveillance Modelling Network^10^ estimates showed similar results of overdiagnosis whether screening was biennial for ages 50–74 or annual for ages 40–74. Post-mortem studies have shown that about 1.3% of women have undetected invasive breast cancer and 8.9% have DCIS, suggesting that cases of DCIS are largely responsible for the overdiagnosis.^19^

The only ethical way to measure overdiagnosis is by using statistics to compare incidence in a screened population with that in an unscreened population. Nonetheless, large estimates of overdiagnosis have been shown to be a function of a failure to correct a bias because of certain statistical confounders, such as lead time and trends in breast cancer risk and incidence over time.^15^

Arleo et al.^10^ showed that screening-detected cancers, including all overdiagnosis cancers, do not disappear spontaneously without excision or treatment. These cancers will be seen at the next screen, and hence they deduced that starting screening at a later age or increasing the screening intervals will delay but not reduce the number of over-diagnosed cancers.

Overtreatment of breast cancers found at screening mammography is of great concern, this being unnecessary surgery, radiation and sometimes even chemotherapy. The so-called overtreatment of patients may be compounded by the side effects of these drastic treatments. This could occur with the diagnosis of an indolent tumour or if a patient dies from another disease after the diagnosis, usually seen in older patients or in patients with comorbidities.

There are no direct ways of measuring which cases will be overtreated. Screening-detected DCIS may or may not lead to invasive cancers, making the selection to treat these difficult. Studies have proven that DCIS diagnosis and treatment has led to a decrease in invasive tumours. Duffy et al.^20^ estimated that for every three cases of DCIS discovered, there was one less case of invasive cancer over a three-year follow-up period.

In reality, it is overtreatment that is the greatest harm of overdiagnosis, with all of the other harms (extra views, sonography and biopsies, as well as the cost and psychological stress involved) being comparatively minor. As such, a diagnosis in itself is not per se the problem; it is the decision to treat every positive diagnosis aggressively, that is more debatable. However, there is currently no known way to decide which lesions at mammography or which histological diagnosis (particularly with invasive disease) would be associated with overtreatment. Furthermore, no disappearance or decrease in size of tumours has been detected in retrospective studies over long screening intervals.^12^

## Screening guidelines

Currently, the screening recommendations for the average-risk woman are indicated for: women with no personal history of breast cancer, no known or suspected BRCA mutation, no previous radiotherapy to the chest, less than a 15% lifetime risk and non-dense breast tissue.^21^

Current screening protocols for an average-risk woman are similar worldwide. No programmes begin screening women before age 50 and few have shorter than 12–18 month screening intervals. Most advocate screening from age 50 and longer screening intervals (24–36 months). The American College of Radiology (ACR) has the longest recommended age range for screening and is the only one recommending annual screening. The UK has the longest recommended screening interval at three years.^22^

The World Health Organization (WHO) recommends two-yearly screening in large, national-based screening programmes. In countries with limited resources in public hospitals (where most patients are diagnosed with advanced cancers), women need to be educated and should ideally have access to screening mammography and appropriate treatment.^23^

South African recommendations have historically been based on American guidelines, some of which changed in 2009 and again in 2016. Until 2009, most of the American societies recommended annual mammography starting from 40. The current guidelines in the United States are as follows:

- The ACR and the National Comprehensive Cancer Institute recommend annual screening from age 40. The stated reasons are that annual screening is associated with the greatest mortality reduction, an increase in life years gained, less chance of advanced disease at diagnosis and better treatment options.^24^
- The American Cancer Society (ACS) suggests annual mammography from 45 to 54 years, biennial studies from 55 and ongoing screening thereafter, depending on the individual’s health. A choice can be made by a woman to have yearly screening from the age of 40 onwards, following a discussion with a healthcare adviser and after being informed of the benefits and harms of screening.^25^
- The American Society of Breast Surgeons has the same recommendations^26^ as the ACS.
- The American College of Obstetricians and Gynaecologists recommends annual or biennial screening mammography from 40 until 75.^27^
- The United States Preventative Services Task Force (USPSTF), updated in 2016,^28^ recommends biennial screening from age 50 to 74. Women from 40 to 49 years can discuss options to screen with their healthcare provider. They believe there is insufficient evidence to support screening in women older than 75.

The factors influencing the United Sates screening guidelines include the following:

- The ACS and USPSTF consider mortality reduction to be the only benefit of screening.
- The USPSTF has adopted the statistic of a 15% mortality reduction from their RCT analysis, albeit using one interpretation of the trials rather than the overall long-term analysis.
- The ACR and ACS both base their guidelines on more recent observational trial data, but these results may underestimate the benefits of screening with modern technology.
- Harms of screening are calculated by the USPSTF and ACS through studies showing the results on population groups and not individuals.
- The ACR and ACS question the accuracy of overdiagnosis rates saying that estimates have not accounted for changing trends in incidence, differences in populations and the presence of differing lead times of cancers found at screening.^29,30^
- The USPSTF recommends that the age of screening starts at 50 and not 40 in order to reduce overdiagnosis. It also recommends biennial screening for the same reason.
- Nonetheless, all the organisations acknowledge the benefits of starting screening mammography at 40 and broadly concur with the results of the CISNET data,^6^ which shows the largest reduction in mortality. They also all recommend allowing women the choice to start screening at age 40 and to end screening at an appropriate time for each person. They are all in agreement about individual discussions with primary healthcare physicians with respect to the benefits and harms of screening, so that each person can make their own decisions.

Quality Control standards are in place in all screening programmes. These should be used to assess accuracy of screening in any given programme. These include an almost universal use of the Breast Imaging Reporting and Data System (BI-RADS) programme, which allows for a set format of reporting and auditing for radiologists.^31^ The current BI-RADS atlas allows scientific assessment as to whether a result of screening is a true-positive (TP), a true-negative (TN), a false-positive (FP) or a false-negative (FN).

The sensitivity (TP / [TP + FN]) of the mammogram exam, the specificity (TN / [TN + FP]) and positive predictive value (TP / [TP + FP]) can then be calculated. Together, these are deemed the Cancer Detection Rate (CDR).

The efficacy of screening is dependent on the recall rate, the CDR and the percentage of early cancers. The CDR has traditionally been approximately 4–6 per 1000 women. The recall rate recommendation has been 10% or less; however, in a recent publication by Grabler et al.,^32^ recall rates of 12% – 14% were found to be associated with more cancer detection. The CDR can be used in assessing screening results, but it has limitations for low-volume practices and radiologists; as low numbers are not statistically significant and therefore cannot accurately assess performance in these situations.^33^

## Screening interval guidelines

Screening intervals should be optimised and this is related to differences in tumour growth. Tumour growth rate is variable and the time taken between initiation of tumour growth and its clinical manifestation is known as the sojourn time. Screening intervals according to Eby^34^ should be influenced by knowing about the heterogeneity of tumours and their sojourn time, and also by the fact that earlier tumour detection would be associated with lower staging and a lower death rate, and in turn lead to lowering treatment costs. The longer the screening interval, the larger the fast-growing tumours will be when detected and these in turn will be associated with later-stage disease.^35^

Biennial mammography is recommended by those who believe that the false positive results can be materially reduced and other harms too can be reduced.^34^ However, according to Destounis et al.,^36^ two-yearly screening in the 40–50 age group is too long, as tumours in this age group have a shorter sojourn time. A study carried out on patients in their 40s, who had annual screening before cancer diagnosis versus those diagnosed symptomatically, showed that those screened had a better chance of survival.

## Screening mammography in the 40–50 years age group

Screening in the 40s is currently not recommended in screening guidelines in most countries. This is because it is thought that the harms outweigh the benefits of screening. Specifically, younger women have denser breast tissue and this leads to higher recall rates, more investigations and more biopsies. However, this false positivity is offset by the fact that tumours reported in younger-aged women are typically more aggressive and have a shorter lead time (of less than two years). Accordingly, the delay of screening in this age group leads to the finding of more and larger later-stage tumours.^37,38,39^

It is generally thought that there is a lower prevalence of cancer at this age. However, it has been shown that there is not much of a difference in cancer incidence between the 40–50 and the 50–60 age groups, as shown in the SEER study of 2009–2013.^40^ In this study, the prevalence of cancer was shown to be 0.3–0.6 per 1000 women between 30 and 39 years; 1.2–1.9 per 1000 women between 40 and 49 years; 2.2–2.6 per 1000 women between 50 and 59 years and 3.4–4.2 per 1000 women between 60 and 69 years.

In 2015, the diagnosis of breast cancer in the United States in the 40s age group was shown to comprise about 17% of the total number of breast cancer diagnoses.^41^ In addition, it has been estimated that 40% of years of life lost will occur in women diagnosed with breasts cancer in the 40–50 year age group.^42^ On the other hand, the early results of RCTs did not show benefit in the 40–50 age group. This has been thought to be because of technical errors and biases as shown in the next paragraph.

In the Two-County Trial, it was shown that there was an increase in the interval cancers (those cancers presenting clinically between screenings) of women in their 40s with only one-view screening and two-year intervals.^1,4^ Furthermore, the Canadian National Breast Screening Study (‘CNBSS’)^13^ did not show any significant decrease in breast cancer mortality in the 40–50-year-old screened group and this was attributed mainly to flaws in the way it was set up.^3,4^

It is thought that the poor results of the RCTs in the 40–49-year-old group were because of:

- Longer interval screening. In this age group, tumours have a shorter sojourn time (time taken for breast cancer from its appearance on imaging to its clinical presentation). In an article by Duffy et al.,^14^ it was found that the mean sojourn time in 40–49 age group was 2.44 years compared with 3.70 years for the 50–59 age group and 4.17 years for the 60–69 age group.
- Inadequate screening technology. Single-view mammography was not good enough in the early days of screening to detect tumours in young, dense breasts.^38^

In 1997, a meta-analysis of long-term follow-ups on the eight RCT trials screening women in the 40–50 age group over 10.5 to 18 years showed:

- A mortality decrease of 18% in the group of women invited to screen.
- A 29% mortality reduction in the five Swedish RCTs.^6^
- A 24% mortality reduction was shown when excluding the CNBSS from the meta-analysis of the eight trials. The CNBSS was excluded as it showed a mortality increase. Critics of this trial say there was a selection bias based on the fact that clinical examinations were carried out and those with disease were selected to be in the screening programme.^7,8^

Coldman et al.,^9^ in the Pan-Canadian Study of Mammography Screening and Mortality from breast cancer between 1990 and 2009, showed a 44% reduction in mortality in the 40–50 age group, compared to those in the 50–59 (40%), 60–69 (42%) and 70–79 (35%) age groups. The USPSTF uses a figure of 15% mortality reduction benefit for women in their 40s, based on the early results of their RCT.^43^

Engel JR et al.^44^ showed a better prognosis in women in their 40s who undertake annual screening. Webb ML et al.^45^ showed those who have interval cancers diagnosed between a two-year screening period have a 47% increase in mortality relative to those screened annually. In the same study, they also demonstrated that in women 50 years and older, the interval cancer detection between two-yearly screening intervals was associated with a 34% lower mortality rate compared to women in the 40–50 age group. Most women prefer annual screening in their 40s and most physicians still recommend screening in this age group.^33^

It has been suggested in all the new guidelines that only those at high risk in the 40–50-year-old age group should undergo screening. However, this would still miss most of the cancers in the age group, as many studies show that most cancers found in this age group are in women of an average-risk profile.^35,46,47,48^

In summary, the data mentioned show unequivocally the benefit of screening in the 40–50-year-old age group, in both the long-term follow-up of RCT and in the numerous observational studies.

## Screening mammography in South Africa

A national breast cancer screening programme in South Africa does not currently exist because of insufficient funds to warrant screening when it is deemed that more important health issues exist. Moreover, there has been little epidemiological research carried out in this field, and as such, South Africa’s statistics of breast cancer incidence in the population may not reflect all the cancers occurring over a given period. Nonetheless, there is a breast cancer registry (at the National Institute for Occupational Health) and current statistics are available on the 2014 data, albeit that these could be incomplete and do not specify whether the cancers were screen detected.^49^

Although the variation in demography is huge in South Africa, it is interesting to note that there was no racial difference in the incidence of breast cancer.^49^ A relatively small percentage of women, generally those in the cities, have access to private medical insurance and are thus funded for screening mammograms. South Africa’s account of statistics is poor, mainly because screening occurs opportunistically in small practices scattered throughout the country. These individual practices generally do not keep audits of screening activity, albeit such statistics could be collected and collated, which is thought by the author and colleagues to be of great benefit to both national and private health providers and to the medical aid funders.

Breast cancer screening and diagnosis is complex. The potential breast cancer diagnosis engenders great fear in many South African women, while information is neither always available nor is it adequately disseminated. It tends to be believed that breast cancer is a uniformly bad diagnosis, and that life-threatening outcomes will be averted by having regular mammograms. In this regard, women have unrealistic expectations of the accuracy of a single mammogram, as well as the benefits and the harms of screening. Furthermore, much screening mammography occurs in regular radiology practices, as opposed to dedicated screening practices that have better facilities. These latter practices are arguably more qualified than others, which women are not necessarily aware of.

Currently, the Radiological Society of South Africa (RSSA) and Breast Imaging Society of South Africa (‘BISSA’) recommend annual screening from 40 to 70 and regular self- and clinical examination. The largest private Medical Aid Group, Discovery Health, currently funds biennial mammography starting from the age of 45 but will fund annual mammography from 40 in those with specified risk factors.

The Cancer Association of South Africa (CANSA) recommends annual screening for women of 40–54 years, and biennial mammography for women of 55 years and older. It also says that it is an individual’s choice to continue annual mammograms from 55 years and that a woman should have the option to continue as long as there is a life expectancy of 10 years or more. The Cancer Association of South Africa also recommends discussion of any breast health problems with primary physicians. It also states that every woman should be provided with data concerning the benefits and harms of screening mammography (CANSA fact sheet April 2017).

## Conclusion

Screening has been shown through studies to reduce the breast cancer mortality rate by 30% – 40%. Current screening guidelines universally endorse screening in the 40–50-year-old age group and it has been proven in the literature that mammography starting from age 40 reduces mortality from breast cancer. Therefore, in light of the general guidelines suggesting that screening should start after 50, women in the 40–50 age group should be given the option to have screening after being made aware of the pros and cons of the screening process. The benefit of regular mammography today is thought to be better than that showed in the early RCT trials. Improved technology, especially with Digital Breast Tomosynthesis (DBT), will enable more screening-detected cancers with less callbacks and biopsies.

Individual screening protocols may be more beneficial than standardised ones and will lead to less morbidity with treatment. Some may be able to have no radiation, some low-fractionated radiation, some adjuvant chemotherapy and, with learning the implications of different tumour biology, less treatment for low-grade DCIS.

In the South African setting, there should be a published guideline clearly defining recommendations for all types of risk groups. Moreover, where access to mammography is difficult domestically, screening by breast examination (performed by nursing staff and field workers), although contentious, may be an option together with ready access to further investigations and treatment. However, this is definitely not the best practice and one should continue recommending screening for all South Africans, this being a situation that in the long run would be most beneficial to all South African women, and would in fact be the most economically sensible one (given the healthcare cost savings from early cancer detection and treatment).
